# Simplex Back Propagation Estimation Method for Out-of-Sequence Attitude Sensor Measurements

**DOI:** 10.3390/s22207970

**Published:** 2022-10-19

**Authors:** Shu Ting Goh, M. S. C. Tissera, RongDe Darius Tan, Ankit Srivastava, Kay-Soon Low, Lip San Lim

**Affiliations:** Department of Electrical & Computer Engineering, National University of Singapore, Singapore 117583, Singapore

**Keywords:** attitude estimation, Kalman filter, out-of-sequence measurement, small satellite

## Abstract

For a small satellite, the processor onboard the attitude determination and control system (ADCS) is required to monitor, communicate, and control all the sensors and actuators. In addition, the processor is required to consistently communicate with the satellite bus. Consequently, the processor is unable to ensure all the sensors and actuators will immediately respond to the data acquisition request, which leads to asynchronous data problems. The extended Kalman filter (EKF) is commonly used in the attitude determination process, but it assumes fully synchronous data. The asynchronous data problem would greatly degrade the attitude determination accuracy by EKF. To minimize the attitude estimation accuracy loss due to asynchronous data while ensuring a reasonable computational complexity for small satellite applications, this paper proposes the simplex-back-propagation Kalman filter (SBPKF). The proposed SBPKF incorporates the time delay, gyro instability, and navigation error into both the measurement and covariance estimation during the Kalman update process. The performance of SBPKF has been compared with EKF, modified adaptive EKF (MAEKF), and moving–covariance Kalman filter (MC-KF). Simulation results show that the attitude estimation error of SBPKF is at least 30% better than EKF and MC-KF. In addition, the SBPKF’s computational complexity is 17% lower than MAEKF and 29% lower than MC-KF.

## 1. Introduction

The development and manufacturing of the small satellite have been a focus in the industry over the past decades. It is reported that approximately 350 small satellites with a mass of less than 200 kg have been launched in 2021 [1]. The Satellite Technology And Research Centre (STAR) at the National University of Singapore is currently developing three satellites named Lumelite-1 to -3 for formation flying programs [2]. Each satellite has a wet mass of 18 kg. The Lumelite satellites are planned to be launched in 2023.

The Lumelite satellite bus system primarily consists of the attitude determination and control subsystem (ADCS), the onboard computer (OBC) system, the electrical power system (EPS), and the communication interface module (CIM). Each of these subsystems is required to continuously monitor its electrical status, communicate with the sensor or actuator that connects to the subsystem, and communicate with OBC. For example, the ADCS’s digital signal processor (DSP) is required to ensure a stable power supply to all sensors, and have no over-current drawn by any actuator, while continuously requesting data from the sensor and sending a command to the actuator at a fixed sampling interval. Due to the reason that each sensor requires a certain processing time upon receiving the request, and the DSP is processing other tasks concurrently the updated data from each sensor and actuator would be received at different time instances, which results in unsynchronized-sensor and actuator information.

Estimation algorithms, such as the extended Kalman filter (EKF) [3], Unscented Kalman filter (UKF) [4,5], Cubature Kalman filter (CKF) [6] and particle filter [7], are commonly used in the attitude determination process. In [8,9], the constraint condition of the quaternion is incorporated into the Kalman gain matrix computation. In [10], a fixed EKF gain method was introduced to lower the nanosatellite’s computational cost. In this case, the KF directly selects the respective gain matrix based on the attitude control mode of the satellite. On the other hand, [11] introduces the square root-based UKF (SRUKF) algorithm with fault detection and identification (FDI) capabilities. The FDI detects and isolates any outlier measurement to ensure the stable performance of the SRUKF algorithm. In addition, [12] introduces the dual vector discrete-time complementary filter (DV-DTCF) where each sensor has its corresponding estimated state vector. Each estimated state vector is updated with respect to its sensor reading; then, all the estimated states are fused in the z-domain via a given transfer function. While numerous estimation algorithms are available in the literature, the sensor data is often assumed to be fully synchronized without delay.

The unsynchronized sensor information, also known as the out-of-sequence measurement (OOSM), would degrade the estimation accuracy. As such, various algorithms have been developed to minimize the accuracy loss. The OOSM problem and associated Kalman filter-based solution were discussed in [13]. Subsequently, [14] presented the algorithm for OOSM-based multi-sensor multi-target application. In [15], the time measurement errors, due to the signal’s traveling time between transmitter and receiver, as well as processing time, have been taken into account when deriving the measurement model for the EKF process. The OOSM-based UKF was implemented in [16] while the PF algorithm was implemented in [17]. Both OOSM-based UKF and PF algorithms are capable of achieving higher estimation accuracy, but these algorithms generally have higher computational costs [18,19]. The weighted measurement fusion method was introduced in [20], with the scalar weight computed based on the distance between the transmitter and receiver. While these algorithms have been developed for unsynchronized sensor data applications, they are primarily developed for target tracking (or positioning) and not for satellite attitude determination applications.

The predictor–observer method was introduced in [21] for attitude estimation with delayed measurements. Its results show that it is capable of converging the attitude estimation error while the EKF experiences error divergence due to the existence of a large sensor delay. The modified adaptive EKF (MAEKF) was proposed in [22] by fusing the N-step of delay measurements and state transition matrices. The MAEKF has much higher estimation accuracy than the re-iterated EKF. However, the methods in [21,22] require additional memory to store N-step delayed measurements. In [23], the moving–covariance Kalman filter (MC-KF), which uses an additional smoothing process to improve the estimation accuracy due to unsynchronized sensor data, has been proposed. It demonstrated the feasibility of orientation estimation, but it is only applied in a two-dimensional position with a one-dimensional angle estimation scenario.

This paper proposed the simplex-back-propagation Kalman filter (SBPKF) to improve the accuracy loss of EKF due to unsynchronized sensor data. The SBPKF utilizes the power series of an exponential matrix to provide a simplification of delayed measurement vector estimation. In addition, the sun vector and magnetometer measurement covariances are formulated with the consideration of additional measurement error due to gyro noise, gyro bias in-stability, and global positioning system (GPS) error. Furthermore, this paper presents the derivation of MC-KF for quaternion and gyro bias estimation. The estimation accuracy of the proposed SBPKF has been benchmarked with EKF, MAEKF [22], and MC-KF in terms of the sensor delay period. The computational complexity of SBPKF, EKF, MAEKF, and MC-KF has also been compared in terms of the total number of multiplications per iteration process.

The paper is organized as follows. Section 2 discusses the ADCS DSP task process. Section 3 presents the standard EKF derivation, and Section 4 presents the MC-KF algorithm. The proposed SBPKF is presented in Section 5. Section 6 presents the simulation and results. Finally, Section 7 concludes the paper.

## 2. Tasks of Attitude Determination and Control System

**Figure 1:**
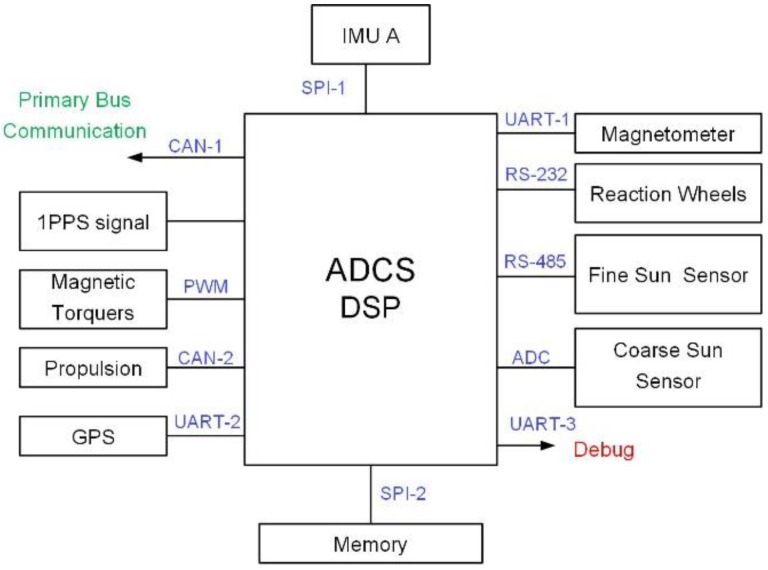
Input and output of attitude determination and control system’s digital signal processor.

Figure 1 illustrates the input and output (I/O) of ADCS between sensors, actuators, satellite bus systems, memory, payload, and DSP. Three Universal Asynchronous Receiver/Transmitter (UART) interfaces are used for the communication between DSP and magnetometer, communication between DSP and GPS receiver, and ADCS debugging purposes. The GPS receiver also directly provides a 1 pulse-per-second (1PPS) signal to DSP via a dedicated interface. The DSP communicates with the satellite bus system via a Controller Area Network (CAN) interface. The additional CAN interface is used for propulsion system communication purposes. The DSP controls the magnetic torquer via pulse-width modulation (PWM) interface, while it receives the coarse sun sensor data via an analog-to-digital converter (ADC) I/O. Lastly, the DSP communicates with a fine sun sensor and reaction wheel via RS-485 and RS-232 interfaces, respectively.

**Figure 2:**
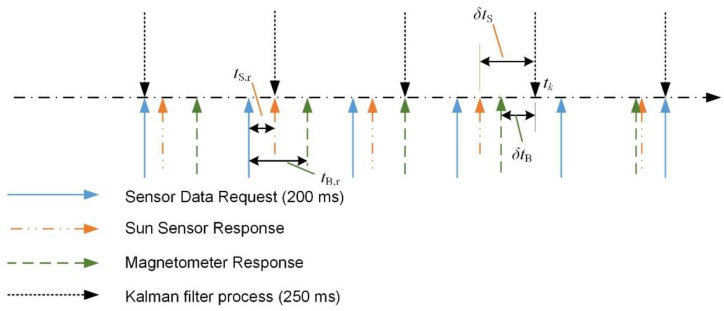
Timestamp of attitude determination task, sensor task, and response of sensor.

The I/O diagram in Figure 1 indicates that the DSP is required to perform various tasks to ensure the stability of the satellite. Furthermore, Table 1 indicates the sampling period of each task that must be handled by the DSP. During the early phase of firmware development and testing, it had been noticed that the sensor data acquisition process requires at least 55 milliseconds to 75 milliseconds (represented by $t_{S,r}$ and $t_{B,r}$ in Figure 2) per cycle. As shown in Figure 2, both the sun sensor and magnetometer require a certain time to respond to the request sent by DSP ($t_{S,r}$ and $t_{B,r},$ respectively) when the sensor task is initiated. For the ideal scenario, $t_{S,r}$ and $t_{B,r}$ shall be less than 5 milliseconds. It is noted that the test process only involves both ADC tasks and sensor tasks. It is expected that the sensor data acquisition process requires much longer time in the full firmware implementation.

In Figure 2, the attitude determination (AD) task and sensor task are performed at different frequencies. In addition to the delay in the sensor data acquisition process, the asynchronization issue between sensor data and the AD task (or $\delta t_{S}$ by sun sensor and $\delta t_{B}$ by magnetometer, respectively) would degrade the overall attitude determination accuracy. Thus, an algorithm to minimize the accuracy degradation due to the sensor data synchronization issue is required. As previously mentioned, the sun sensor or magnetometer measurement will only be considered as a delayed measurement if either $\delta t_{S}$ or $\delta t_{B}$. is more than five milliseconds. The five milliseconds is the expected response time of the sensor under the ideal operation scenario.

## 3. Extended Kalman Filter

The small satellite is typically equipped with sun sensors, magnetic torquers, and microelectromechanical systems (MEMS) based inertial measurement unit (IMU). The MEMS IMU device includes both gyroscope and magnetometer sensors. The output of the MEMS IMU gyroscope is given by [24]

(1) $${\tilde{\omega }}_{k}=\omega_{k}+\beta +\eta_{g}+\eta_{\beta }$$

where overhead notation of “$\tilde{.}$” denotes measurement, ${\tilde{\omega }}_{k}$ denotes the measured three-axis body rate, $\omega_{k}$ denotes the truth three-axis body rate, $\eta_{g}$ denotes the output noise of the gyroscope, β denotes the truth gyroscope bias, and $\eta_{\beta }$ denotes the gyroscope bias due to the angular random walk and bias instability.

The general EKF process consists of gain matrix computation, estimated state and covariance update, and estimated state and covariance propagation. For EKF-based attitude determination, the objective is to estimate both the satellite’s attitude (or quaternion) and gyroscope bias. Thus, the estimated state vector is ${\hat{x}}_{k}=\left[\begin{array}{cc} {\hat{\varrho }}_{k}^{T} & {\hat{\beta }}^{T} \end{array}\right]$. The $\varrho_{k}$ is the vector component of the quaternion vector, q, such that [25]

(2) $${\hat{q}}_{k}=\left[\begin{array}{cc} {\hat{\varrho }}_{k}^{T} & {\hat{q}}_{4,k} \end{array}\right]$$

where the overhead notation of “$\hat{.}$” denotes the estimated vue, and ${\hat{q}}_{4,k}$ is the scalar of the quaternion vector with ${\hat{q}}_{4,k}=\sqrt{1-{\hat{\varrho }}_{k}^{T}{\hat{\varrho }}_{k}}$.

Given a pair of measurement vectors from sensors, and their corresponding estimated measurement vector, both ${\hat{q}}_{k}$ and $\hat{\beta }$ are updated via the following process [25]

(3) $${\hat{q}}_{k}^{+}={\hat{q}}_{k}^{-}+0.5\Xi \left({\hat{q}}_{k}^{-}\right)\delta \hat{\varrho }\;{\hat{\beta }}^{+}={\hat{\beta }}^{-}+\delta \hat{\beta }$$

where $\Xi \left(q_{k}\right)$ is defined in [25] and

(4) $$\left[\delta \begin{array}{cc} {\hat{\varrho }}_{k}^{T} & \delta {\hat{\beta }}^{T} \end{array}\right]=K\left(\tilde{y}-{\hat{y}}_{k}\right)$$

In (4), $y\equiv {\left[\begin{array}{cc} y_{S}^{T} & y_{B}^{T} \end{array}\right]}^{T}$, where the subscript “S” denotes the sun vector and subscript “B” denotes the earth’s magnetic field vector. In addition, the superscript “−” denotes pre-update, superscript “+” denotes post update, and K denotes the Kalman filter gain matrix [26]

(5) $$K=P_{k}^{-}H_{k}^{T}{\left(H_{k}P_{k}^{-}H_{k}^{T}+R_{k}\right)}^{-1}$$

where $R_{k}$ denotes measurement noise covariance and $H_{k}$ denotes the Jacobian matrix of measurement vectors. Moreover, the mth sensor component of the estimated measurement vector in (4) can be expressed as [25]

(6) $${\hat{y}}_{m}=A\left({\hat{q}}_{k}^{-}\right){\hat{e}}_{m}$$

where $A\left({\hat{q}}_{k}^{-}\right)$ denotes the estimated attitude matrix at time $t_{k}$, and ${\hat{e}}_{m}$ (a unit vector) is the estimated mth sensor’s measurement in the earth center inertia (ECI) reference frame. It is noted that the derivation of ${\hat{e}}_{S}$ associated to ${\hat{y}}_{S}$ is detailed in [27], and ${\hat{e}}_{B}$ is provided in Appendix A and (31)**.** In addition, $H_{k}$ in (5) is given as [26]

(7) $$H_{k}=\left[\begin{array}{cc} \partial {\hat{y}}_{S}/\partial \varrho & \partial {\hat{y}}_{S}/\partial \beta \\ \partial {\hat{y}}_{B}/\partial \varrho & \partial {\hat{y}}_{B}/\partial \beta \end{array}\right]$$

For standard EKF, $\partial {\hat{y}}_{S}/\partial \varrho$, $\partial {\hat{y}}_{B}/\partial \varrho$, $\partial {\hat{y}}_{S}/\partial \beta$ and $\partial {\hat{y}}_{B}/\partial \beta$ are defined as [28]

(8) $$\partial {\hat{y}}_{S}/\partial \varrho =\left[{\hat{y}}_{S}\times \right]\;\partial {\hat{y}}_{B}/\partial \varrho =\left[{\hat{y}}_{B}\times \right]\;\partial {\hat{y}}_{S}/\partial \beta =\partial {\hat{y}}_{B}/\partial \beta =0_{3\times 3\;}$$

Here, $\left[a\times \right]$ is the cross-product matrix [25]. By assuming that there is no cross-correlation between the sun vector and the earth magnetic field vector, $R_{k}$ can be written as

(9) $$R_{k}=\left[\begin{array}{cc} R_{S,k} & 0\\ 0 & R_{B,k} \end{array}\right]$$

where $R_{S,k}$ is the covariance associated with the sun sensor, and $R_{B,k}$ is the covariance associated with the magnetometer.

In general, the measurement vector, ${\tilde{b}}_{m}$ output by the *m^th^* sensor in the satellite body reference frame always contains an error, with the standard deviation of $\nu_{m}$:

(10) $${\tilde{b}}_{m}=b_{m}+\nu_{m}$$

where $b_{m}$ denotes the truth measurement reading of the *m^th^* sensor. Furthermore, ${\tilde{b}}_{m}$ maybe not necessarily a unit vector (e.g., Earth’s magnetic field vector). Therefore, ${\tilde{y}}_{m}$ represents the normalized vector of ${\tilde{b}}_{m}$ such that ${\tilde{y}}_{m}={\tilde{b}}_{m}/\|{\tilde{b}}_{m}\|$, where ‖.‖ denotes the vector’s magnitude. Then, the general expression of $R_{m,k}$ in (9) with the consideration of vector normalization is given as [29]:

(11) $$R_{m,k}={\bar{R}}_{m,k}+\frac{1}{2}trace\left({\bar{R}}_{m,k}\right)\frac{{\tilde{b}}_{m}{\tilde{b}}_{m}^{T}}{{\left\|{\tilde{b}}_{m}\right\|}^{2}}$$

(12) $${\bar{R}}_{m,k}=\frac{-{\left[{\tilde{b}}_{m}\times \right]}^{2}}{∥{\tilde{b}}_{m}{∥}^{3}}E\left\{v_{m}v_{m}^{T}\right\}{\left(\frac{-{\left[{\tilde{b}}_{m}\times \right]}^{2}}{∥{\tilde{b}}_{m}{∥}^{3}}\right)}^{T}$$

with $E\left\{.\right\}$ denotes expected value, $trace\left({\bar{R}}_{m,k}\right)$ denotes the summation of diagonal elements of ${\bar{R}}_{m,k}$. In addition, the state error covariance update is given as [26]

(13) $$P_{k}^{+}=\left(I-KH_{k}\right)P_{k}^{-}$$

Using the following definition, ${\hat{\omega }}_{k}={\tilde{\omega }}_{k}-\hat{\beta }$, the nonlinear quaternion propagation model is given as [30]

(14) $$\dot{q}=\frac{1}{2}\bar{\Omega }\left({\hat{\omega }}_{k}\right){\hat{q}}_{k}^{-}$$

where $\bar{\Omega }\left({\hat{\omega }}_{k}\right)$ is defined in [30]. Due to the nature of quaternion multiplication, the integration of (14) is often highly complex. Instead, the quaternion propagation can be simplified using the N_x_-step summation model [31]:

(15) $${\hat{q}}_{k+1}^{-}\approx \left(I_{4\times 4\;}+\overset{N_{x}}{\underset{n=1}{\sum }}\frac{1}{n!}{\bar{\Omega }}^{n}\left({\tilde{\omega }}_{k}\right){\left(\Delta t_{k}\right)}^{n}\right){\hat{q}}_{k}^{+}$$

The state error covariance propagation model is given by [26]

(16) $$\dot{P}=F_{k}P_{k}^{+}+P_{k}^{+}F_{k}^{T}+Q$$

where [25]

(17) $$F_{k}=\left[\begin{array}{cc} -\left[{\tilde{\omega }}_{k}-\hat{\beta }\times \right] & -I_{3\times 3}\\ 0 & 0 \end{array}\right]\;Q=E\left\{\eta_{g}\eta_{g}^{T}\right\}+E\left\{\eta_{\beta }\eta_{\beta }^{T}\right\}$$

The standard EKF shows that the state vector update process in (3) to (5), and the Jacobian matrix in (8) do not consider the unsynchronized sensor data problem. On the other hand, the proposed SBPKF and MC-KF include the sensor data delay and its associated sensitivity matrix and error covariance in the measurement model estimation. The MC-KF algorithm is presented in the next Section.

## 4. Moving Covariance Kalman Filter

The presented MC-KF in this section is based on [23,32]. It has a similar estimation process as DV-DTCF in [12] but a different state vector fusion approach. Let us define the following time instance, $t_{m}=t_{k}-\delta t_{m}$, where subscript “m” is a general representation of either sun sensor,“S” or magnetometer “B”. First, given the sun sensor and magnetometer data arrive at different time instances, there are two sets of state correction vector, $\Delta {\hat{x}}_{m,t_{m}}$

(18) $$\Delta {\hat{x}}_{m,t_{m}}=K_{m}\left({\tilde{y}}_{m}-{\hat{y}}_{m,t_{m}}\right)=\left[\delta \begin{array}{cc} {\hat{\varrho }}_{m,t_{m}}^{T} & \delta {\hat{\beta }}_{m,t_{m}}^{T} \end{array}\right]$$

where $K_{m}$ is the Kalman filter gain matrix that is derived with respect to the sensitivity matrix of each sensor:

(19) $$K_{m}=P_{k}^{-}H_{m,t_{m}}^{T}{\left(H_{m,t_{m}}P_{k}^{-}H_{m,t_{m}}^{T}+R_{m,k}\right)}^{-1}$$

where $R_{m,k}$ is defined in (11).

From (18), two sets of updated state vectors, ${\hat{x}}_{m,{\;t}_{m}}^{+}$ (or ${\hat{x}}_{S,{\;t}_{S}}^{+}$ and ${\hat{x}}_{B,{\;t}_{B}}^{+}$) and two sets of state error covariances, $P_{m,{\;t}_{m}}^{+}$(or $P_{S,{\;t}_{S}}^{+}$ and $P_{B,{\;t}_{B}}^{+}$) will be obtained by using a similar formulation in (3) and (13). Next, both ${\hat{x}}_{S,{\;t}_{S}}^{+}$, $P_{S,{\;t}_{S}}^{+}$ and ${\hat{x}}_{B,{\;t}_{B}}^{+}$, $P_{B,{\;t}_{B}}^{+}$ pairs are required to be synchronized and smoothed. First, let’s define the following time instance, $t_{u}$ based on the following sensor delay information

(20) $$t_{u}\equiv \left\{\begin{array}{cc} \;t_{k} & \delta t_{S}\leq 5\;\mathrm{ms}\;\mathrm{and}\;\delta t_{B}\leq 5\;\mathrm{ms}\\ t_{k}-\delta t_{S} & \delta t_{S}\geq \delta t_{B}\\ t_{k}-\delta t_{B} & \delta t_{B}\geq \delta t_{S} \end{array}\right.$$

**Figure 3:**
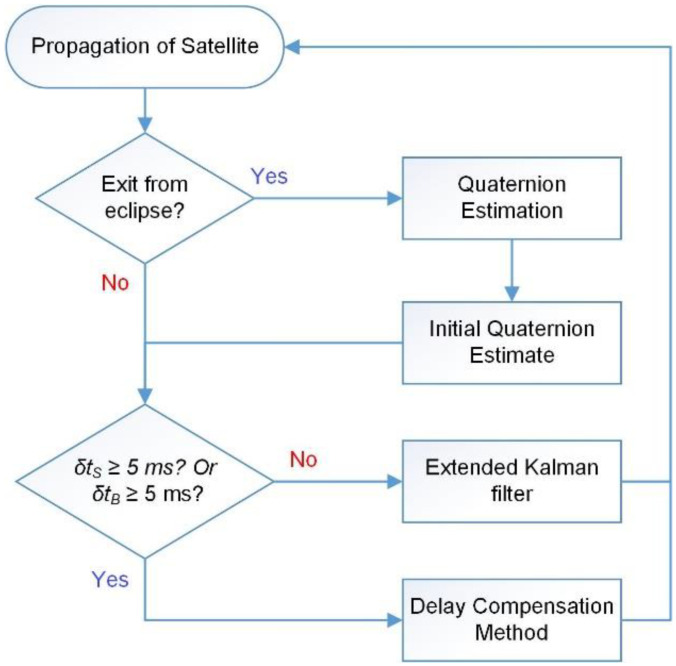
Algorithm flow diagram for delayed sensor scenario.

As presented in Figure 3, the delay of 5 ms is assumed to be the minimum time required by a sensor to instantly reply to the sensor data request by ADCS DSP. Then, the ${\hat{x}}_{P,{\;t}_{p}}$ and $P_{P,{\;t}_{p}}$ pair that has a lesser delay with respect to $t_{k}$, are propagated to $t_{u}$

(21) $${\hat{x}}_{P,{\;t}_{p}}^{+}\to {\hat{x}}_{P,{\;t}_{u}}^{-}$$

(22) $$P_{P,{\;t}_{p}}^{+}\to P_{P,{\;t}_{u}}^{-}$$

For generalization purposes, the sensor data with a higher delay is labeled as w*^th^* sensor. Based on [23], we define the following matrices, $P_{P/W}$ and $P_{P-W}$

(23) $$P_{P-W}=P_{P,t_{u}}^{-}{\left(K_{W}H_{W}\right)}^{T}+P_{W,{\;t}_{u}}^{+}{\left(K_{P,t_{u}}H_{P,t_{u}}\right)}^{T}+5E-4\times \mathrm{diag}\left(P_{P,t_{u}}^{-}+P_{W,{\;t}_{u}}^{+}\right)$$

(24) $$P_{P/W}=P_{P,t_{u}}^{-}{\left(K_{W}H_{W}-K_{P,t_{u}}H_{P,t_{u}}\right)}^{T}$$

where $K_{m,t_{u}}$ and $H_{m,t_{u}}$ are Kalman filter gain and sensitivity (derivation see (7), (8), (33) and (34)) matrices at $t_{u}$. To ensure the positive definite of $P_{P-W}\;$ matrix, we have

(25) $$P_{P-W}^{*}=P_{P-W}+5E-4\times \mathrm{diag}\left(P_{P,t_{u}}^{-}+P_{W,{\;t}_{u}}^{+}\right)$$

In (25), “diag(A)” denotes the matrix only contains the diagonal elements of matrix A. The purpose is to ensure the matrix $P_{P-W}^{*}$ is invertible. Then, the smoothed state vector is given as

(26) $${\hat{x}}_{t_{u}}^{+}={\hat{x}}_{P,t_{u}}^{-}+P_{P/W}{\left(P_{P-W}^{*}\right)}^{-1}\;\delta x_{t_{u}}$$

where

(27) $$\delta x_{t_{u}}={\hat{x}}_{W,t_{u}}^{+}-{\hat{x}}_{P,t_{u}}^{-}$$

The quaternion update corresponding to (26) is given in (3). On the other hand, the quaternion subtraction to compute $\delta x_{t_{u}}$ is given as follows:

(28) $${\hat{q}}_{W,t_{u}}^{+}-{\hat{q}}_{P,t_{u}}^{-}\equiv \left[\begin{array}{cc} \Psi \left({\hat{q}}_{W,t_{u}}\right) & {{\hat{q}}_{W,t_{u}}}^{+} \end{array}\right]\left[\begin{array}{c} -\varrho_{P}^{-}\\ q_{P,4}^{-} \end{array}\right]$$

with $\Psi \left(q\right)$ is defined in [25]. Finally, the covariance is smoothed as follows

(29) $$P_{t_{u}}^{+}=P_{P,t_{u}}^{-}-P_{P/W}{\left(P_{P-W}^{*}\right)}^{-1}P_{P/W}$$

Lastly, both state vectors, ${\hat{x}}_{t_{u}}^{+}$ and covariance, $P_{t_{u}}^{+},$ are propagated to the next time step, ${\hat{x}}_{k+1}^{-}$ and $P_{k+1}^{-}\;$ via (15) and (16).

## 5. Simplex-Back-Propagation Kalman Filter

The proposed SBPKF uses the similar Kalman filter process as EKF, but with different measurement and covariance models. The SBPKF has taken the delay between sensor data acquisition time and Kalman sampling time, $\delta t_{m}$ (or known as $\delta t_{S}$ and $\delta t_{B}$ in Figure 2). As such, the delayed measurement vector for both the sun sensor and magnetometer in (6) is rewritten as:

(30) $$y_{m,t_{k}-\delta t_{m}}=\left(I_{3\times 3\;}+\overset{N_{y}}{\underset{n=1}{\sum }}\frac{{\left(-1\right)}^{n}}{n!}{\left(\left[{\tilde{\omega }}_{k}-\hat{\beta }\times \right]\delta t_{m}\right)}^{n}\right)A\left({\hat{q}}_{k}^{-}\right){\hat{e}}_{m}$$

It is noted that the Earth’s magnetic field vector in the ECI reference frame, $e_{B}$ is computed based on the satellite position and velocity at $t_{k}-\delta t_{B}$

(31) $$e_{B}=A_{\mathrm{NED}}^{\mathrm{ECI}}B\left(r_{t_{k}-\delta t_{B}},v_{t_{k}-\delta t_{B}}\right)$$

where $A_{\mathrm{NED}}^{\mathrm{ECI}}$ denotes the transformation matrix of NED to ECI reference frame, and $B={\left[\begin{array}{ccc} B_{\theta } & B_{\phi } & B_{r} \end{array}\right]}^{T}$ denotes the Earth’s magnetic field vector with each component representing North, East, and Down direction, respectively. For simplicity, the derivation of $A_{\mathrm{NED}}^{\mathrm{ECI}}$ will not be shown in this paper, but the associated algorithm and Matlab code are available in [33]. In addition, $r_{t_{k}-\delta t_{B}}$ and $v_{t_{k}-\delta t_{B}}$ can be approximated as

(32) $$r_{t_{k}-\delta t_{B}}=r_{t_{k}-}+\delta t_{B}v_{t_{k}}-\frac{\mu r_{t_{k}}\delta t_{B}^{2}}{2\|r_{t_{k}}\|{}^{3}}\;v_{t_{k}-\delta t_{B}}=v_{t_{k}-}-\frac{\mu r_{t_{k}}\delta t_{B}}{2\|r_{t_{k}}\|{}^{3}}$$

From (30), the measurement vector is in terms of both quaternion and gyro bias. Therefore, both $\partial {\hat{y}}_{S}/\partial \beta$ and $\partial {\hat{y}}_{B}/\partial \beta$ are no longer zero matrices. Instead, with the additional summation terms, (8) becomes

(33) $$\partial {\hat{y}}_{m}/\partial \varrho =\left(I_{3\times 3\;}+\overset{N_{y}}{\underset{n=1}{\sum }}\frac{{\left(-1\right)}^{n}}{n!}{\left(\left[{\tilde{\omega }}_{k}-\hat{\beta }\times \right]\delta t_{m}\right)}^{n}\right)\left[{\hat{y}}_{m}\times \right]$$

(34) $$\frac{\partial {\hat{y}}_{m}}{\partial \beta }=-\left[{\hat{y}}_{m}\times \right]\delta t_{m}+\frac{\delta t_{m}^{2}}{2}\left(\left[{\tilde{\omega }}_{k}-\hat{\beta }\times \right]\left[{\hat{y}}_{m}\times \right]+\left[\left[{\tilde{\omega }}_{k}-\hat{\beta }\times \right]{\hat{y}}_{m}\times \right]\right)$$

The general expression of $R_{m,k}$ remains the same as in (11). However, (31) indicates the satellite positioning error contributes to the Earth’s magnetic field modeling error. In addition, both $\eta_{g}$ and $\eta_{\beta }$ contribute additional errors in (30). Therefore, both ${\bar{R}}_{S,k}$ and ${\bar{R}}_{B,k}$, which were originally formulated in (12) have been modified to become:

(35) $${\bar{R}}_{S,k}=\frac{-{\left[{\tilde{b}}_{S}\times \right]}^{2}}{\|{\tilde{b}}_{S}\|{}^{3}}\left(E\left\{\nu_{S}\nu_{S}^{T}\right\}+{\hat{R}}_{S,\delta t_{S}}\;\right){\left(\frac{-{\left[{\tilde{b}}_{S}\times \right]}^{2}}{\|{\tilde{b}}_{S}\|{}^{3}}\right)}^{T}\;{\bar{R}}_{B,k}=\frac{-{\left[{\tilde{b}}_{B}\times \right]}^{2}}{\|{\tilde{b}}_{B}\|{}^{3}}\left(E\left\{\nu_{B}\nu_{B}^{T}\right\}+{\hat{R}}_{B,\delta t_{B}}+\sigma_{B}\left(r_{t_{k}-\delta t_{B}}\right)\;\right){\left(\frac{-{\left[{\tilde{b}}_{B}\times \right]}^{2}}{\|{\tilde{b}}_{B}\|{}^{3}}\right)}^{T}$$

where

(36) $${\hat{R}}_{m,\delta t_{m}}=\Lambda_{m}Q\Lambda_{m}^{T}+\frac{\delta t_{m}^{4}}{4}trace\left(Q\right)\left({\hat{y}}_{m}{\hat{y}}_{m}^{T}Q+Q{\hat{y}}_{m}{\hat{y}}_{m}^{T}+trace\left(Q\right){\hat{y}}_{m}{\hat{y}}_{m}^{T}\right)$$

(37) $$\Lambda_{m}\equiv \left[{\hat{y}}_{m}\times \right]\delta t_{m}-\frac{\delta t_{m}^{2}}{2}\left[{\tilde{\omega }}_{k}-\hat{\beta }\times \right]\left[{\hat{y}}_{m}\times \right]-\frac{\delta t_{m}^{2}}{2}\left[\left[{\tilde{\omega }}_{k}-\hat{\beta }\times \right]{\hat{y}}_{m}\times \right]$$

It is noted that the term $\sigma_{B}\left(r_{t_{k}-\delta t_{B}}\right)$ denotes additional error covariance due to the satellite position error:

(38) $$\sigma_{B}\left(r_{t_{k}-\delta t_{B}}\right)=A_{\mathrm{NED}}^{\mathrm{ECI}}\frac{\partial B}{\partial h_{\mathrm{LLA}}}\frac{\partial h_{\mathrm{LLA}}}{\partial r_{\mathrm{ECEF}}}E\left\{\eta_{r}\eta_{r}^{T}\right\}{\left(A_{\mathrm{NED}}^{\mathrm{ECI}}\frac{\partial B}{\partial h_{\mathrm{LLA}}}\frac{\partial h_{\mathrm{LLA}}}{\partial r_{\mathrm{ECEF}}}\right)}^{T}$$

In (38), $\partial B/\partial h_{\mathrm{LLA}}$ denotes the partial derivative of $B\left(r_{t_{k}-\delta t_{B}},v_{t_{k}-\delta t_{B}}\right)$ in (31) with respect to longitude, ϕ, latitude, θ and radius, r (or $h_{\mathrm{LLA}}={\left[\begin{array}{ccc} \theta & \phi & r \end{array}\right]}^{T}$). In addition, $\partial h_{\mathrm{LLA}}/\partial r_{\mathrm{ECEF}}$ denotes the partial derivative of conversion from position in the Earth-centered-Earth-fix (ECEF) reference frame to longitude, latitude, and radius. The formulation for $\partial B/\partial h_{\mathrm{LLA}}$ is available in Appendix A, and the formulation for conversion of ECEF position to longitude, latitude, and radius is detailed in [34].

The overall process flow for SBPKF (and MC-KF/MAEKF) is presented in Figure 3. When the satellite exits from the eclipse, the first pair of sun and Earth magnetic field vectors will be input into the quaternion estimation (QUEST) algorithm [35] to provide an initial quaternion vector, with the assumption of fully synchronized measurement vectors. The quaternion output from QUEST also guarantees the stability of EKF and SBPKF algorithms as the EKF-based algorithm is often susceptible to initial condition error [36]. As discussed in Section II, the respective measurement will only be considered as a delayed measurement for the filtering process if $\delta t_{m}>5\;\mathrm{ms}$. Thus, if both $\delta t_{S}$ and $\delta t_{B}$ are less than a given threshold (i.e 5 ms), standard EKF will be conducted. Otherwise, the delayed measurement vector in (30) will be computed. Once the measurement covariances are computed using (11), (35) to (37), the SBPKF updates the estimated states and state error covariance using the (3) and (13).

## 6. Simulation and Results

**Figure 4:**
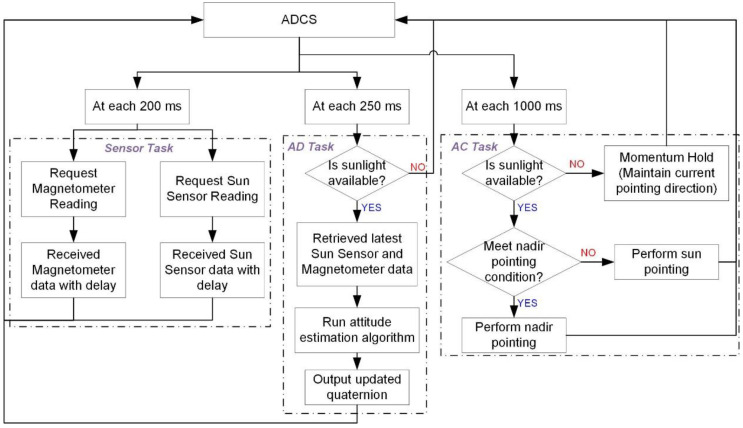
Process flow of sensor task, attitude determination (AD) task, and attitude control (AC) task for attitude determination Monte Carlo simulation.

**Figure 5:**
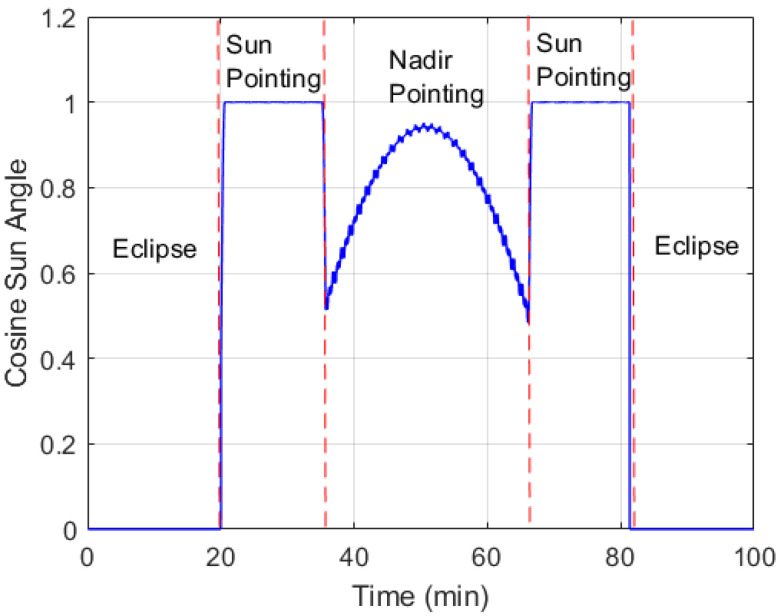
Satellite attitude control condition for Monte Carlo simulation.

Monte Carlo simulations have been conducted to compare the quaternion and gyro bias estimation accuracy for the proposed SBPKF, EKF, MAEKF in [22], and MC-KF, with respect to sensor delay. The Monte Carlo simulation environment is illustrated in Figure 4. Three ADCS tasks are considered in the simulation, which are the sensor task, AD task, and AC task. The sensor task is simulated at a sampling rate of 200 ms, with selected sensor delays. The sensor delay to be range from 65 ms to 145 ms, with its standard deviation given in Table 2. The AC task simulates the truth quaternion vector that is corresponding to the satellite pointing profile in Figure 5. The AD task comprises the attitude determination algorithm, such as SBPKF, EKF, MAEKF, and MC-KF. During the eclipse period (or without sunlight), the satellite attitude control enters the momentum hold condition. The default attitude control of the satellite during the sunlight condition is the sun-pointing mode. The satellite performs nadir pointing when the angle between the nadir axis and sun vector in the satellite body frame is within the sun sensor’s half-cone field-of-view (or 60 degrees). In Figure 5, the satellite begins to perform nadir pointing 15 min after entering the sunlight condition, for approximately 35 min.

For each Monte Carlo simulation, the performance of SBPKF, EKF, MAEKF, and MC-KF are evaluated for one sunlight period (or approximately 60 min). The satellite orbital parameters are provided in Table 2. It is noted that the right ascension of ascending node, the argument of perigee, and the initial true anomaly are randomly generated in each Monte Carlo simulation.

The sensors and gyro noises, GPS accuracy, sensor, and EKF sampling rate are listed in Table 2. The sun sensor, GPS, gyro, and magnetometer noises are based on the commercial off-the-shelf product, with the consideration of noise density and sensitivity. In addition, the configuration of MAEKF is based on details provided in [22].

### 6.1. Accuracy Comparison

**Figure 6:**
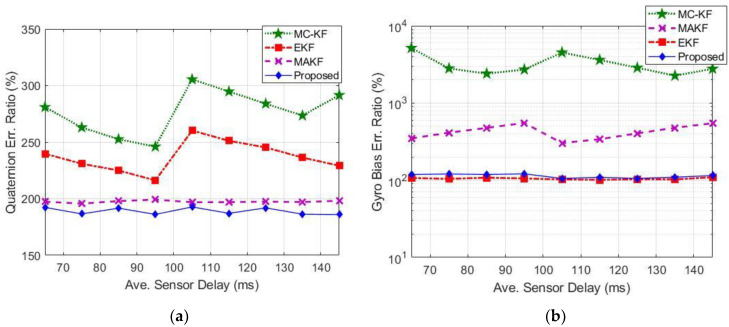
Estimation accuracy performance comparison with respect to zero-sensor delay scenario: (**a**) Magnitude of quaternion error, and (**b**) Magnitude of gyro bias error.

Figure 6 compares the quaternion and gyro bias estimation error between the SBPKF, EKF, MAEKF, and MC-KF with respect to the estimation results without the sensor delay scenario. The average quaternion error magnitude and gyro bias error magnitude for the zero sensor delay scenario is 0.145 degrees and 2.8 millidegrees, respectively. The quaternion error ratio, $\overset{=}{q},$ and gyro bias error ratio, $\overset{=}{\beta },$ in Figure 6 are given by:

(39) $$\overset{=}{q}=\frac{\sum_{k}^{M_{d}}\|\sin^{-1}∆q_{k,d}\|}{\sum_{k}^{M_{0}}\|\sin^{-1}∆q_{k},0\|}\frac{M_{0}}{M_{d}}\times 100\%$$

(40) $$\overset{=}{\beta }=\frac{\sum_{k}^{M_{d}}\|∆\beta_{k,d}\|}{\sum_{k}^{M_{0}}\|∆\beta_{k,0}\|}\frac{M_{0}}{M_{d}}\times 100\%$$

where $M_{d}$ is the total number of samples for the delayed sensor scenario, $M_{0}$ is the total number of samples for no sensor delay scenario, $∆q_{k}$ is the quaternion error, and $∆\beta_{k}$ is gyro bias error at *k^th^* sample with subscript “d” representing delayed sensor, scenario, and the subscript “0” representing no sensor delay scenario, respectively.

Figure 6a shows that the SBPKF has the lowest quaternion error magnitude, followed by MAEKF. The SBPKF’s quaternion estimation error ratio is approximately 189% (or 0.274 degrees) when compared to the no-sensor-delay scenario. The MAEKF quaternion error ratio is approximately 200% (or 0.29 degrees). Figure 6a also shows that without the simplex-back-propagation method, the quaternion estimation error ratio of EKF is increased to 220–250% (or 0.319 to 0.363 degrees), and the quaternion estimation error ratio of MC-KF is increased to 250–300% (or 0.363 to 0.435 degrees).

Figure 6b shows that the EKF has the lowest gyro bias error magnitude. However, the gyro bias error magnitude difference between EKF and the SBPKF can be considered negligible as both are in the range of millidegrees (or approximately three millidegrees). On the other hand, the MAEKF gyro bias error is three times higher than the SBPKF, even though it has a similar quaternion estimation error as the SBPKF. The additional error that occurs in MAEKF could be due to the reason that the MAEKF assumes all sensor data received at the same time instance. However, in practice, each sensor has a different time delay.

Although the MC-KF is designed to compensate for the error due to data delay, the introduction of unknown gyro bias as the initial condition has greatly degraded the performance of the MC-KF. From Figure 6b, the results show that the MC-KF has a large gyro bias error ratio as compared to the SBPKF and EKF. The MC-KF’s gyro bias error is approximately five times higher than the scenario without data delay. The MC-KF uses one measurement vector to update each ${\hat{x}}_{S,{\;t}_{S}}$ and ${\hat{x}}_{B,{\;t}_{B}}$ (see (18) and (26)). However, the attitude determination process requires at least a pair of measurement vectors to effectively estimate the quaternion vector. Furthermore, the additional covariance update process in (29) causes the state error covariance to be underestimated in MC-KF. The study in [37] has shown that an additional covariance update process within an iteration without a proper smoothing procedure could degrade the estimation accuracy. Therefore, the results in Figure 6 show that MC-KF would require a more complex derivation and implementation to ensure a stable estimation performance.

On the other hand, the proposed SBPKF considers the time delay of sensor data, $\delta t_{m}$ when estimating the measurement vector in (30). In addition, (35) to (38) consider the additional propagation error due to the measurement model used in (30). Thus, the underestimation of error covariance is avoided, and the estimation accuracy is improved.

Overall, the results in Figure 6 provide the ADCS performance guideline during the system design review stage. The expected quaternion estimation error increment from Figure 6 allows the evaluation if an additional sensor, such as a star tracker is required to meet the system design requirement. STAR centre is presently developing a 50 kg microsatellite with a star tracker for high-precision pointing applications. The applicability of SBPKF will be verified on the engineering model. Subsequently, its in-orbit attitude determination accuracy performance will be benchmarked with the quaternion computed by the star tracker.

### 6.2. Computational Cost

Table 3 compares the number of multiplications required by EKF, SBPKF, MAEKF, and MC-KF at each iteration. The number of multiplication required for matrix multiplication and an inverse matrix is based on the method in [20]. The MAEKF’s multiplication number is the average multiplication number per sampling step within one second. This is due to the reason that the number of available measurement pairs for MAEKF varies between 1 and 2 at each sampling step.

For the SBPKF, MAEKF, and MC-KF, we assume the scenario where δt>5 ms. Table 3 shows that the number of multiplications required by MC-KF and MAEKF is approximately two times higher than EKF. The number of multiplication required by SBPKF is 17% lesser than MAEKF and 29% lesser than MC-KF, respectively. Although the SBPKF requires 65% more multiplication compared to EKF, the quaternion estimation error is improved by 30 to 45% with a similar gyro bias estimation error. Thus, the SBPKF achieves a higher attitude estimation accuracy at an expense of higher computational complexity.

## 7. Conclusions

This paper presented the simplex-back-propagation Kalman filter (SBPKF) for delayed sensor data-based quaternion and gyro bias estimation. The estimated measurements are formulated with the consideration of sensor data’s time delay to improve the quaternion estimation accuracy. In addition, the noise covariance matrices are derived with the presence of navigation error, gyro bias, and gyro noises to prevent underestimating the estimation error. The detailed implementation of the moving–covariance Kalman filter (MC-KF) for quaternion estimation has been presented. Monte Carlo simulations have been conducted to compare the accuracy and computational complexity of the proposed SPBKF, extended Kalman filter (EKF), modified adaptive EKF (MAEKF), and MC-KF. The results show that the SBPKF average estimated quaternion error is at least 30% lower than both EKF and MC-KF. Although the MAEKF quaternion estimation error is slightly higher than the SBPKF, its gyroscope bias estimation error is three times higher than SBPKF. In addition, the SPBKF computational complexity is 17% better than MAEKF and 29% better than MC-KF.

For future work, the SPBKF will be evaluated using the engineering model of a 50 kg microsatellite. The impact of the digital signal processor’s 8-bit floating point on the overall SPBKF accuracy performance will also be investigated.

## Figures and Tables

**Table 1:** ADCS DSP task.

Sampling Period	Task
100 ms	ADC Task
200 ms	Sensor Task
250 ms	AD task
500 ms	Housekeeping Task
1000 ms	AC task

**Table 2:** Simulation Configuration.

Parameter	Value	Parameter	Value
Semimajor axis	6963.145 km	Sun sensor noise, $\nu_{S}$	0.1 deg
Eccentricity	0.0001	Magnetometer noise, $\nu_{B}$	45 nT
Inclination	10 deg	Gyro noise, $\eta_{g}$	0.135 deg
$N_{x}$	3	Gyro bias, β	±0.2 deg/s
EKF sampling rate	4 Hz	In-run bias stability, $\eta_{\beta }$	4.8456 mdeg/hr
Sensor sampling rate	5 Hz	Position Error, $\eta_{r}$	15 m
Sensor Delay Standard Deviation	5 ms		

**Table 3:** Comparison of number of multiplications.

Algorithm	Number of Multiplications
EKF	4104
Proposed	6774
MAEKF	8066
MC-KF	9406

## Appendix A

This appendix provides an overview of the derivation of the Earth’s magnetic field and its associate partial derivative. From Appendix H in [38], the North–East–Down components of the Earth’s magnetic field vector (or $B_{\theta }$, $B_{\phi }$ and $B_{r}$) are given as

(A1) $$B_{\theta }=-\overset{k}{\underset{n=1}{\sum }}{\left(\frac{a}{r}\right)}^{n+2}\overset{n}{\underset{m=0}{\sum }}\left(g^{n,m}\cos m\phi +h^{n,m}\sin m\phi \right)\frac{\partial P^{n,m}\left(\theta_{c}\right)}{\partial \theta_{c}}\;B_{\phi }=-\frac{1}{\sin\theta_{c}}\overset{k}{\underset{n=1}{\sum }}{\left(\frac{a}{r}\right)}^{n+2}\overset{n}{\underset{m=0}{\sum }}m\left(-g^{n,m}\sin m\phi +h^{n,m}\cos m\phi \right)P^{n,m}\left(\theta_{c}\right)\;B_{r}=-\overset{k}{\underset{n=1}{\sum }}{\left(\frac{a}{r}\right)}^{n+2}\left(n+1\right)\overset{n}{\underset{m=0}{\sum }}\left(g^{n,m}\cos m\phi +h^{n,m}\sin m\phi \right)P^{n,m}\left(\theta_{c}\right)$$

Here, $\theta_{c}$ is the geocentric co-latitude such that

(A2) $$\theta_{c}=90-\theta$$

where θ is the geodetic latitude.

The partial derivate of each component in B, with respect to the North–East–Down direction (or known as θ, ϕ, and r, respectively) are given as

(A3) $$\frac{\partial B_{\theta }}{\partial \theta }=\overset{k}{\underset{n=1}{\sum }}{\left(\frac{a}{r}\right)}^{n+2}\overset{n}{\underset{m=0}{\sum }}\left(g^{n,m}\cos m\phi +h^{n,m}\sin m\phi \right)\frac{\partial^{2}P^{n,m}\left(\theta_{c}\right)}{\partial \theta_{c}^{2}}\;\frac{\partial B_{\phi }}{\partial \theta }=\frac{1}{\sin\theta_{c}}\overset{k}{\underset{n=1}{\sum }}{\left(\frac{a}{r}\right)}^{n+2}\overset{n}{\underset{m=0}{\sum }}m\left(-g^{n,m}\sin m\phi +h^{n,m}\cos m\phi \right)\frac{\partial P^{n,m}\left(\theta_{c}\right)}{\partial \theta_{c}}\;+\frac{\cos\theta_{c}}{\sin^{2}\theta_{c}}\overset{k}{\underset{n=1}{\sum }}{\left(\frac{a}{r}\right)}^{n+2}\overset{n}{\underset{m=0}{\sum }}m\left(-g^{n,m}\sin m\phi +h^{n,m}\cos m\phi \right)P^{n,m}\left(\theta_{c}\right)\;\frac{\partial B_{r}}{\partial \phi }=-\overset{k}{\underset{n=1}{\sum }}{\left(\frac{a}{r}\right)}^{n+2}\left(n+1\right)\overset{n}{\underset{m=0}{\sum }}m\left(-g^{n,m}\sin m\phi +h^{n,m}\cos m\phi \right)P^{n,m}\left(\theta_{c}\right)$$

(A4) $$\frac{\partial B_{r}}{\partial \theta }=\overset{k}{\underset{n=1}{\sum }}{\left(\frac{a}{r}\right)}^{n+2}\left(n+1\right)\overset{n}{\underset{m=0}{\sum }}\left(g^{n,m}\cos m\phi +h^{n,m}\sin m\phi \right)\frac{\partial P^{n,m}\left(\theta_{c}\right)}{\partial \theta_{c}}\;\frac{\partial B_{\theta }}{\partial \phi }=-\overset{k}{\underset{n=1}{\sum }}{\left(\frac{a}{r}\right)}^{n+2}\overset{n}{\underset{m=0}{\sum }}m\left(-g^{n,m}\sin m\phi +h^{n,m}\cos m\phi \right)\frac{\partial P^{n,m}\left(\theta_{c}\right)}{\partial \theta_{c}}\;\frac{\partial B_{\phi }}{\partial \phi }=-\frac{1}{\sin\theta_{c}}\overset{k}{\underset{n=1}{\sum }}{\left(\frac{a}{r}\right)}^{n+2}\overset{n}{\underset{m=0}{\sum }}-m^{2}\left(g^{n,m}\cos m\phi +h^{n,m}\sin m\phi \right)P^{n,m}\left(\theta_{c}\right)$$

(A5) $$\frac{\partial B_{\theta }}{\partial r}=\left(n+2\right)\overset{k}{\underset{n=1}{\sum }}\frac{a^{n+2}}{r^{n+3}}\overset{n}{\underset{m=0}{\sum }}\left(g^{n,m}\cos m\phi +h^{n,m}\sin m\phi \right)\frac{\partial P^{n,m}\left(\theta_{c}\right)}{\partial \theta_{c}}\;\frac{\partial B_{\phi }}{\partial r}=\frac{\left(n+2\right)}{\sin\theta_{c}}\overset{k}{\underset{n=1}{\sum }}\frac{a^{n+2}}{r^{n+3}}\overset{n}{\underset{m=0}{\sum }}m\left(-g^{n,m}\sin m\phi +h^{n,m}\cos m\phi \right)P^{n,m}\left(\theta_{c}\right)\;\frac{\partial B_{r}}{\partial r}=\left(n+2\right)\overset{k}{\underset{n=1}{\sum }}\frac{a^{n+2}}{r^{n+3}}\left(n+1\right)\overset{n}{\underset{m=0}{\sum }}\left(g^{n,m}\cos m\phi +h^{n,m}\sin m\phi \right)P^{n,m}\left(\theta_{c}\right)$$

It is noted that based on (A2), the partial derivate of the Earth’s magnetic field with respect to geodetic latitude θ in (A3) to (A5) has been simplified based on the following definition

(A6) $$\frac{\partial B}{\partial \theta }\equiv \frac{\partial B}{\partial \theta_{c}}\frac{\partial \theta_{c}}{\partial \theta }=-\frac{\partial B}{\partial \theta_{c}}$$

In addition, from Appendix H in [38], the Gauss function $P^{n,m}\left(\theta_{c}\right)$ are given as below:

(A7) $$P^{0,0}\left(\theta_{c}\right)=1$$

(A8) $$P^{n,n}\left(\theta_{c}\right)=\sin\theta_{c}P^{n-1,n-1}\left(\theta_{c}\right)$$

(A9) $$P^{n,m}\left(\theta_{c}\right)=\cos\theta_{c}P^{n-1,m}\left(\theta_{c}\right)-K_{P}^{n,m}P^{n-2,m}\left(\theta_{c}\right)$$

(A10) $$K_{P}^{n,m}\equiv \left\{\begin{array}{cc} \frac{{\left(n-1\right)}^{2}-m^{2}}{\left(2n-1\right)\left(2n-3\right)} & \mathrm{if}\;n>1\\ 0 & \mathrm{if}\;n=1 \end{array}\right.$$

The first and second order partial derivative with respect to $\theta_{c}$ can be obtained via (A7) to (A9).
